# Control of hospital acquired infections and antimicrobial resistance in Europe: the way to go

**DOI:** 10.1007/s10354-018-0676-5

**Published:** 2019-01-08

**Authors:** Alex W. Friedrich

**Affiliations:** Department of Medical Microbiology, University Medical Center Groningen, University of Groningen, Groningen, The Netherlands

**Keywords:** Healthcare associated infections, Antimicrobial resistance, Prevention-economics, Transmission, Network medicine

## Abstract

One of the major challenges for modern medicine is our ageing society and an increased level of immunocompromised hosts. More invasive and intensive medical interventions will increase the number of healthcare-associated infections (HCAI), which means infection that occur because of or in concomitance, but in any case, during or after healthcare interventions. Such infections are caused usually endogenously from microbial components of the patient’s own microbiome. Usually, the microorganisms of the microbiome show a natural resistance against a few antibiotics. Due to selection processes and epidemic transmission of specific clones, microorganisms that have become resistant to multiple antibiotics become part of the patient’s microbiome and can subsequently cause infections that are difficult or even impossible to treat. The kind of infections that will occur depends on diverse factors. Already today, according to Cassini et al., 2,609,911 new cases of HCAI occur every year in the European Union and European Economic Area (EU/EEA). The cumulative burden of the six HAIs was estimated at 501 disability-adjusted life years (DALYs) per 100,000 general population each year in the EU/EEA. In a recent publication, 426,277 healthcare-associated infections caused by antimicrobial resistant microorganisms were calculated to occur in the EU every year. Attributable deaths in the EU due to antimicrobial resistant microorganisms were estimated to be 33,110 per year. We know that we cannot prevent all HCAI. Because medical innovations will allow for an increased number of novel treatments that will comprise abiotic materials, microorganisms will adapt to this environment and enhance the risk for new HCAI. The challenge for the future will not be to try to prevent all infections, as some of them will remain unavoidable, but to prevent the occurrence of non-treatable microorganisms that would make unavoidable infections additionally untreatable. That means that we need to reflect on how we organize infection prevention, diagnostics and control. While patients with classical infectious diseases present with infectious diseases (ID)-specific symptoms, patients with HCAI present usually with another underlying disease. HCAI are therefore perceived as a secondary damage not following classical clinical and epidemiological rules. However, more recently we have to consider how we should react to HCAI and antimicrobial resistance (AMR) as they are quite different in epidemiology and transmission behavior than classical infectious diseases. Today, the prevalence of AMR is rising all over Europe. Although good success has been seen in many countries, methicillin-resistant *Staphylococcus aureus* (MRSA) remains an important challenge for many countries. In addition to MRSA, multidrug-resistant *Escherichia coli* and carbapenem-resistant *Enterobacteriaceae* are becoming a problem of public health importance. Furthermore, we need to focus more on implementation of known infection prevention measures than trying to solve the problem by observing and describing it. However, in addition to medical factors such as antibiotic use, hand hygiene etc., we tend to forget that there are factors behind these factors that have a major influence and are found in the structures of our different healthcare systems. We need to look more at the context before we try to implement prevention measures and need to learn from each other. A common goal to tackle carbapenem-resistant *Enterobacteriaceae* (CRE) by 2030 would be an important step to foster collaboration across Europe. As the current funding and remmuneration system does not sufficiently support prevention of HCAI and AMR, it is time for the development of a less production- but more prevention-economic financing system for clinical microbiology and infection control.

## Introduction

Healthcare-associated infections (HCAI) due to microorganisms that are antimicrobial resistant are today one of the most important challenges for modern medicine. Up to 2,609,911 new cases of HCAI occur every year in the European Union and European Economic Area (EU/EEA) [1]. The cumulative burden of the six HCAIs was estimated at 501 disability-adjusted life years (DALYs) per 100,000 general population each year in EU/EEA [1]. In a recent publication, 426,277 healthcare-associated infections caused by antimicrobial resistant microorganisms were calculated to occur in the EU every year [2]. Attributable deaths in the EU due to antimicrobial resistant microorganisms were estimated to be 33,110 per year [2]. At the same time, prevalence of antimicrobial resistance (AMR) is rising all over Europe. Although, good success has been reported for many countries, methicillin-resistant *Staphylococcus aureus* (MRSA) remains an important challenge Europe-wide [2]. In addition to MRSA, multidrug-resistant *Escherichia coli* [2] and carbapenem-resistant *Enterobacteriaceae* are rapidly becoming a problem of public health importance [3]. It is necessary to clarify that there is an important difference in the transmission dynamics between microorganisms causing classical infectious diseases and multidrug resistant microorganisms (MDRO). Different from classical infectious diseases (e. g. measles, tuberculosis, malaria), HCAI due to antibiotic resistant microorganisms do not present a defined incubation period, shedder or carrier time, nor do they follow the natural transmission routes (airborne or contact-droplet) as found in the textbooks. This is because the medical environment as well as novel medical interventions form infection sources and transmission ways that have not been described before (e. g. endoscopes, air-flow, new medical devices, structural merging of clinical departments). Furthermore, in classical infectious disease epidemiology one can draw rather unambiguous conclusions in case of an outbreak looking at person-, time- and place-related data with a specific disease. In a world of HCAI and AMR, time, place and person data are just not enough to understand the epidemiology. In addition to the three parameters, data of the causative microorganisms on a species or better on a subspecies level (molecular or genetic subtype) is needed to be able to define an outbreak and understand the transmission route, which enables to identify efficient intervention points for infection control.

To make it very clear, MDRO are hardly transmittable, unless certain factors coincide at the same time. The most important difference lies e. g. in the disposition factors for AMR causing HCAI. The disposition is in general a combination of susceptibility of the host and the virulence of the respective microorganism. While in classical infectious diseases the exposition is usually exogenous and caused by obligate pathogens for humans, HCAI are often caused endogenously by mesophilic, facultative pathogenic microorganisms. The proper disposition for an infection is therefore in first instance not only depending on the immune system, but on two additional factors. The disposition of such a mesophilic facultative pathogenic microorganism is not only to reach the skin/mucosa of another host, but first to exogenously colonize the new host. This sounds trivial, but is a crucial prerequisite that is often forgotten. It means that it needs to convert from a sort of short-term contamination to long-term colonization. However, the conversion into long-term colonization is not fully understood, but in addition to traditional risk factors for an infection determined by host factors such as surgical wounds, foreign bodies as well as the virulence of the microorganism (e. g. adhesins, type III secretion systems), seem to be important risk factors. These include an “inflammation prone” micro-environment (e. g. ulcers, atopic dermatitis), but in first instance the selection pressure due to antibiotics with sub-inhibitory concentrations mostly on mucosal surfaces.

The second important step is to cause an endogenous infection. For this, healthcare-related factors such as implants and insertion of biofilm-forming medical devices, invasive medical interventions and a changing micro-environment are important risk factors. Only beyond these two steps, i. e. conversion to long-term colonization and intervention-related infection, the lack of a proper immune system becomes relevant for a subsequent invasive infection. This means that pure exposure to AMR is one important *conditio sine qua non*, but not a direct risk. In a world where exposure to AMR cannot be avoided any more, focus should lie on prevention of long-term colonization and subsequent invasive or non-invasive infection. However, in modern healthcare, all exposure and disposition steps accumulate and can create a “perfect storm” for the transmission of AMR as well as subsequent occurrence of HCAI. Under these man-made conditions, MDROs become in fact transmittable from human to human.

How to respond to all this? One way is to develop novel antibiotics, not knowing whether there are still enough new antibiotic mechanisms to be detected, but knowing that introduction of new antibiotics in the past mainly triggered new resistance again. The second approach is to focus on avoiding long-term colonization of humans at the wrong moment, when they have plenty of risk factors for developing an infection. Here, avoiding the spread of specific MDRO becomes a preventive step to maintain prevalence beneath the epidemic threshold. Often it is believed that especially hand and/or environmental hygiene and/or single-room isolation in combination with personal protective equipment can solve the problem. This is unfortunately not the case. Our data and experience show that a solution lies far beyond and requires multiple and complex interventions as discussed below.

### Prevention goal

As the major preventive goal is to *prevent infections and maintain optimal antimicrobial treatmen*t, we consequently need to prevent all avoidable HCAI but especially keep the non-avoidable infections at least treatable. This is possible through preventing the spread of MDRO as well as nonorganism-based AMR avoiding long-term colonization of the human population. Otherwise, a situation could be reached were we will see non-avoidable and non-treatable HCAI. Something we must not allow to occur.

## There is a fire in our house

Imagine that there is a fire in the house you are living in, in one or two of the apartments. Would not everybody be alerted and start helping our neighbor to stop the fire. We would know that the fire will reach our own apartment, too. Even if we would try to close our doors and prefer not to look at the apartments in fire. Instead, in Europe today with respect to AMR, the latter seems happening. The fire stands for multi-drug resistant organisms (MDRO), namely methicillin-resistant *Staphylococcus *aureus (MRSA), vancomycin-resistant enterococci (VRE), but especially carbapenemase-producing microorganisms belonging to the *Enterobacteriaceae* or to non-fermenting gram-negative bacteria, such as *Acinetobacter baumannii* or *Pseudomonas aeruginosa*. They have reached the hospitals of our European Union and are spreading in our neighboring countries while we are looking at it and continue describing the problem or are just trying to produce new antimicrobial agents. I strongly believe that we should start implementing prevention activities at all levels of healthcare in all countries. We should start doing it by mutual and multi-disciplinary collaboration following the real-life transmission ways. This would require a new awareness of the need to prevent AMR and non-treatable HCAI by all medical fields and increase public awareness in all countries.

## Europe carbapenem-resistant *Enterobacteriaceae* (CRE)-free by 2030

The Netherlands and Scandinavian countries are often considered to be an example for other countries, at least when it comes to the prevention of AMR. In fact, looking at the prevalence of multi-drug resistant microorganisms those countries have been for the last 15 years green islands in Europe. At the end of the 1990s MRSA and VRE were the only major MDRO threats in Europe and the Netherlands did very well in the eyes of the rest of Europe.

In 2005, the situation had changed. Since 2010, the Netherlands registers colonization and invasive infections with extended-spectrum beta-lactamases (ESBL) producers (mainly *Escherichia coli* and *Klebsiella pneumoniae*) as well as large outbreaks due to vancomycin-resistant enterococci (VRE). We could show that already 1–6% of all patients admitted to care institutions are colonized with VRE or ESBL [4]. Especially ESBL prevalence has an impact on the empirical use of antibiotics in healthcare institutions and could be one plausible explanation for the gradual increase in the use of carbapenems (NethMap 2016) in the Netherlands since 2004. The cumulative direct selection pressure favouring carbapenem resistance is increasing. Recent and pan-European data by Grundmann et al. [3] showed that in most European countries interregional distribution is already ongoing and in several Southern and Eastern European countries an endemic level has been reached. But also countries as the Netherlands report sporadically occurring outbreaks, almost always due to imports from abroad (e. g. after holidays or transfer from patients from abroad). Allowing an endemic level of carbapenem-resistant microorganisms (CR-MO, i. e. CRE and CR-nonfermenters) would dramatically worsen the treatment options for patients. The practical use of essential new antibiotics is still too far in the future and antibiotics remain an uncertain factor because of new resistance. Because of the above mentioned, the most important preventive task is a “zero tolerance policy” for the occurrence of nosocomial infections caused by MDRO and CR-MO. This asks to prevent the import, colonization or further spread of this group of bacteria. The aim is to keep part of Europe in the year 2030 still green with respect to invasive CR-MO infections that are contracted in the country itself. At the same time, we will need to strengthen efforts in other European countries with medium- and high-level prevalence of CR-MO and start a roll-back intervention.

## Roll-back strategy: invert your outbreak management

In an endemic situation, aiming for a decrease in prevalence needs another approach than the one we have been following for the last decade, focusing on describing the problem more that on acting against it. This is especially true in the countries where MDRO have become endemic. Of course, microbiological diagnostics needs to be performed, not only for preventive screening, but especially from clinical material, as in practice many MDRO are identified during healthcare. This means that clinical microbiological analysis and results become prerequisite. In any case, MDRO should not be transmitted from patient to patient under standard healthcare conditions and extended infection control measures need to be implemented. Hospitals should therefore make a decision and focus on their most important CR-MO, i. e. the strain that is most endemic in their specific hospital and region. This needs to be done by subtyping to establish which CR-MO subtype will be taken into the focus with the goal to decrease prevalence of this specific strain within the next few months or years. For all the other strains, standard procedure continues. This kind of inverted outbreak management needs bi- or multilateral collaboration between expert groups across Europe.

## Ad hoc outbreak-specific diagnostics

To be successful we need to invert the known process of our outbreak management. Today, in case of a suspected outbreak, screening is started among presumably exposed contact-patients, so we identify carriers performing microbiological diagnostics on clinical specimens, which stops at identification at species level comprising the resistance pattern. Because of the un-specificity of this procedure we find not only the outbreak strain, but also other strains and depending on the method used even other MDRO bacteria. As a consequence, we need to react with infection control measures in patients carrying other MDRO and are forced to subtype the strains of the same species and the same resistance pattern. Depending on the technology, method and laboratory used for typing, one gets the information of who epidemiologically belongs to the outbreak and who does not days to weeks later. This procedure is far too cumbersome, slow and not acceptable any more. Instead of screening first, then identify and then type, we should first type using whole genome sequencing and develop *ad hoc* a diagnostic test (e. g. multiplex-PCR[Polymerase Chain Reaction]) that identifies on a subspecies-level only the strain responsible for the outbreak at this very moment. This can realistically be done today within five working days. Once the outbreak specific-PCR has been obtained, patients can be screened in each healthcare institution on a large scale and because of the high specificity on a subspecies level, even pooling of clinical swabs from different patients (e. g. from same room) and iterative screening (e. g. twice a week) is feasible and cost-efficient. Furthermore, the ad-hoc test can be provided to other laboratories in the each healthcare region, so they can specifically search for the outbreak strain. The major advantage is that there is no screening-related “bycatch” anymore, no other, sporadic CR-MO, no ESBL, no VRE that would trigger additional preventive measures and could lead to “infection-control fatigue” in the clinical departments. After the roll-back program has been successful, the region decides on the next most important endemic/outbreak strain to be tackled and so on. This could also be the way for high-endemic countries to start decreasing the prevalence of CR-MO which is often a conglomerate of different epidemic strains circulating in a region or whole country.

## Regional AMR-prevention networks: the hubs make the difference

We usually believe that MDRO are transmitted from humans to humans and transmission needs to be stopped. But in reality, the most important transmission way for MDRO is just following the movement of the host. This means that MDRO are in reality mainly spreading by following their carriers within the healthcare system. With every admission to a care institution, transfer of MDRO can then be triggered under the above-mentioned circumstances. Because patients are transferred within and between healthcare institutions all the time, spread can only be prevented if joint interventions are realized in the whole healthcare region and by all institutions. Additionally, different healthcare systems have different interconnectedness with subsequent differences in patient mobility. The structure of the system itself enables more or less transmission. A calculation of the care network allows for a prediction of the epidemiologically most important hubs and most efficient collaboration forms between hospitals [5]. In the Netherlands, we calculated AMR-prevention regions on the basis of patient transfer between hospitals [5] and in 2016 the Ministry of Health implemented ten AMR-prevention regions, based upon the network analysis of patient transfer patterns. Because healthcare is subject to constant change, all concentration, specialization, fusion as well as contractual changes can have an effect on the healthcare network and thereby influence the risk of spreading MDRO in a country and beyond. The way in which the most effective preventive interventions can be implemented differs per care network. Data shows that one or more “hub hospitals” can be found in every care region [5]. Due to their position in the network, the hubs have a special role in the identification and the fight against AMR. Hubs are often more willing to build and maintain sustainable network structures. However, the network reality shows that other than pure medical factors seem to have major impact on the occurrence and spread of MDRO.

## Look at the context

If other factors than medical ones have influence on the prevalence of MDRO, this might be also true for the use of antibiotics, the implementation of hand hygiene or the microbiological diagnostic frequency. In fact, cross-border studies show that there are major structural differences between healthcare systems of different countries [6]. Comparison between the Dutch and the German healthcare system with respect to structures influencing the prevention of MDRO shows that there are not only more hospitals and hospital beds on the German side (3.6 in NL vs. 8.1 in DE per 1000 inhabitants) [7], but especially much more ICU beds (6.3 in NL vs. 29.2 in DE per 100,000 inhabitants) [8], more doctors having their own practice (0.4 in NL vs. 1.6 in DE per 1000 inhabitants) and finally also more stationary patient cases (9.8 in NL vs. 21.5 in DE per 100 inhabitants) [7]. This has influence on the population exposed to risk factors for the acquisition of MDRO as well as on the exposure to antibiotics. If more people undergo surgery, more people can get a surgical site infection and receive antibiotics. The Dutch hospitals use a mean 60% of the hospital beds whereas German hospitals use more than 80% of their beds [9]. This latter can have major influence on isolation capacity and transmission probability. Furthermore, the ratio between health care worker (HCW) and patients on Dutch intensive care units (ICUs) is usually 1:1 up to 1:1.5, while on the German side normal ratio is 1:2 and reaches in some cases up to 1:3.6. This latter can have major impact on the hand hygiene compliance. Finally, there is a difference in the microbiological diagnostic frequency between the two countries, as in Germany most hospitals have outsourced their diagnostics and need to pay for each analysis, although it might be for preventive reasons. As the majority of Dutch hospitals have their own microbiological laboratory or are associated to a regional microbiological lab, more diagnostics—e. g. in case of an outbreak—does not lead to substantial extra costs, as infrastructure and personnel are already available and consumable costs are rather low for culture-based screenings. This facilitates the performance of preventive microbiological screenings and diagnostics. These are just a few examples showing that structural factors of healthcare systems can have major impact on the medical factors that are usually considered to be the reason for an increase in AMR and MDRO.

## Integrative stewardship: the art of becoming metacompetent

Listening to our major stakeholder, the patient, we know he/she is asking three simple questions: (1) How are you going to protect me today against an infection? (2) Do I have an infection and if so, what is the cause? (3) How can I be optimally treated? The medical professionals who can give optimal answer to these three questions should strive for interdisciplinary collaboration to deliver an integrative stewardship, a trias of (1) infection prevention stewardship (IPS), (2) diagnostic stewardship (DGS) and (3) antimicrobial stewardship (AMS) [10]. It is important to conclude that none of the classically existing monovalent medical specialist fields, such as Infectious Disease, Medical Microbiology or Hygiene are fully able to cover all knowledge and expertise necessary in today’s complexity of AMR and HCAI in large centers. On the other hand, in regular hospitals, a specialist would be needed who is polyvalent and covers some of all three expertises. This means that after having reached the level of being competent in the own expert field we need to go beyond our “own” specialization and department in order to become metacompetent within a larger collaborative organization specific for the patient groups in the local or regional working environment. In the end, as MDROs know no border, European training of professionals is necessary to show that different interventions are possible for the same goals in different countries and healthcare systems. We need to learn how to implement prevention within different socio-economic backgrounds and within different healthcare structures. The European Committee on Infection Control of the European Society of Clinical Microbiology and Infectious Disease (ESCMID) initiated in 2018 the first two-year course on Infection Prevention and Control [11]. This training includes specifically the development of European competence and metacompetence in infection prevention and control.

## Prevention-economic model

For historical reasons, today’s financing system of Medical Microbiology and Infection Control is mainly based on a production-economic model in most countries. Interestingly, although in addition to diagnostics, two more core activities, such as infection prevention (diagnostics) and antimicrobial stewardship, have become important pillars in daily work, but are currently handled as overhead costs and are usually not funded by the health insurance systems. This has several disadvantages. Frequency of microbiological diagnostics is low as it is often seen as an unnecessary cost. In many healthcare refunding systems, it is even economically advantageous for a diagnostic lab, when there are many MDRO outbreaks because of an increase in lab production activity, leading even in some settings to a positive—at least economic—incentive for more MDRO.

Alternative costing models are needed to promote quality of care while at the same time stimulating innovation and efficiency. This includes e. g.

- regional prevention budgets (system allowance) for the regional AMR-prevention networks, based on clear quality criteria,
- a financial incentive for rapid diagnostic in acute-care hospitals, i. e. the faster a result is produced, the more economic it is and not as it is at the moment that the faster a test the higher the cost for the laboratory,
- prevention-fostering reimbursement following an insurance model whereby healthcare institutions could insure the losses of production during an outbreak (e. g. full refunding of a blocked bed due to isolation). This insurance would require specific prerequisites comprising clinical microbiology, infection control expertise, screening, hand hygiene, etc. A risk assessment based on the risk profile (healthcare infections, hand hygiene, etc.) provides a continuous financial incentive for quality as it would have an influence on the premium to be paid by the healthcare institution. With rising numbers of outbreaks and low infection prevention performance, the premium for the hospital rises in the following year.
- Integrated cost models for diagnostics within the price of the antibiotics. Just like in oncological chemotherapy of breast cancer, which can only be reimbursed if the herceptin receptor has been determined in advance. The same could be implemented for certain infections (e. g. urinary tract infection, sepsis, pneumonia), so that antibiotic therapy can only be financed when combined with diagnostics, as antibiotic chemotherapy cannot be performed without having analyzed its effectiveness in parallel. This would require further development of rapid and on-site diagnostic tools.

## Conclusion

Instead of continuing to describe the problem, we need to focus on the context given by each healthcare system before we try to implement prevention measures which means that we need to learn from each other in order to be able to start acting to roll-back AMR where it has become endemic. As refunding presently seems to fail in giving support to prevention of HCAI and AMR, it is time for the development of a less production- but more prevention-economic financing system for clinical microbiology and infection control. A common goal to tackle carbapenem-resistant *Enterobacteriaceae* (CRE) by 2030 would be an important step to foster collaboration across Europe.
